# GvmR – A Novel LysR-Type Transcriptional Regulator Involved in Virulence and Primary and Secondary Metabolism of *Burkholderia pseudomallei*

**DOI:** 10.3389/fmicb.2018.00935

**Published:** 2018-05-16

**Authors:** Linh Tuan Duong, Sandra Schwarz, Harald Gross, Katrin Breitbach, Falko Hochgräfe, Jörg Mostertz, Kristin Eske-Pogodda, Gabriel E. Wagner, Ivo Steinmetz, Christian Kohler

**Affiliations:** ^1^Friedrich Loeffler Institute of Medical Microbiology, University Medicine Greifswald, Greifswald, Germany; ^2^Interfaculty Institute of Microbiology and Infection Medicine Tübingen, University of Tübingen, Tübingen, Germany; ^3^Department of Pharmaceutical Biology, Pharmaceutical Institute, Eberhard Karls University of Tübingen, Tübingen, Germany; ^4^German Centre for Infection Research, Partner Site Tübingen, Tübingen, Germany; ^5^Competence Center Functional Genomics, Junior Research Group Pathoproteomics, University of Greifswald, Greifswald, Germany; ^6^Institute of Hygiene, Microbiology and Environmental Medicine, Medical University of Graz, Graz, Austria

**Keywords:** *Burkholderia pseudomallei*, LTTR, virulence, metabolism, T3SS3, T6SS1

## Abstract

*Burkholderia pseudomallei* is a soil-dwelling bacterium able to survive not only under adverse environmental conditions, but also within various hosts which can lead to the disease melioidosis. The capability of *B. pseudomallei* to adapt to environmental changes is facilitated by the large number of regulatory proteins encoded by its genome. Among them are more than 60 uncharacterized LysR-type transcriptional regulators (LTTRs). Here we analyzed a *B. pseudomallei* mutant harboring a transposon in the gene BPSL0117 annotated as a LTTR, which we named *gvmR* (globally acting virulence and metabolism regulator). The *gvmR* mutant displayed a growth defect in minimal medium and macrophages in comparison with the wild type. Moreover, disruption of *gvmR* rendered *B. pseudomallei* avirulent in mice indicating a critical role of GvmR in infection. These defects of the mutant were rescued by ectopic expression of *gvmR*. To identify genes whose expression is modulated by GvmR, global transcriptome analysis of the *B. pseudomallei* wild type and *gvmR* mutant was performed using whole genome tiling microarrays. Transcript levels of 190 genes were upregulated and 141 genes were downregulated in the *gvmR* mutant relative to the wild type. Among the most downregulated genes in the *gvmR* mutant were important virulence factor genes (T3SS3, T6SS1, and T6SS2), which could explain the virulence defect of the *gvmR* mutant. In addition, expression of genes related to amino acid synthesis, glyoxylate shunt, iron-sulfur cluster assembly, and syrbactin metabolism (secondary metabolite) was decreased in the mutant. On the other hand, inactivation of GvmR increased expression of genes involved in pyruvate metabolism, ATP synthesis, malleobactin, and porin genes. Quantitative real-time PCR verified the differential expression of 27 selected genes. In summary, our data show that GvmR acts as an activating and repressing global regulator that is required to coordinate expression of a diverse set of metabolic and virulence genes essential for the survival in the animal host and under nutrient limitation.

## Introduction

*Burkholderia pseudomallei* is the causative agent of melioidosis, a frequently fatal infectious disease affecting humans and animals which occurs predominantly in Northern Australia, Southeast Asia, China, and Taiwan. However, melioidosis cases and environmental isolates of *B. pseudomallei* have been reported from several regions worldwide between latitude 20°N and 20°S (Inglis et al., 2006; McRobb et al., 2014; Currie, 2015). Common clinical presentations are acute pneumonia and sepsis that are associated with high mortality rates even after appropriate antibiotic treatment (Wiersinga et al., 2006; Limmathurotsakul and Peacock, 2011). Various underlying diseases, such as diabetes mellitus, chronic renal failure, and chronic lung disease are risk factors for developing melioidosis (Limmathurotsakul and Peacock, 2011). As an environmental pathogen *B. pseudomallei* is adept at surviving and proliferating in diverse environments such as the soil and mammalian and non-mammalian hosts (Wiersinga et al., 2018). This ability has been ascribed to its large genome and its extensive repertoire of virulence factors such as type 3 secretion systems (T3SS), type 6 secretion systems (T6SS), and regulatory proteins (Holden et al., 2004). The genome of the *B. pseudomallei* type strain K96243 is predicted to encode more than 20 two-component sensor-regulator systems, 20 sigma (s) factors including extracytoplasmic function (ECF) s factors, more than 60 LysR-type transcriptional regulators (LTTRs) and other classes of regulatory proteins (Holden et al., 2004). However, the function and regulon of the vast majority of these regulators currently remain unknown.

LTTRs are ubiquitous among bacteria and potentially comprise the largest family of prokaryotic transcription factors (Perez-Rueda and Collado-Vides, 2001; Maddocks and Oyston, 2008). They contain a N-terminal helix-turn-helix (HTH) DNA binding domain and function as dual regulators that can activate or repress gene expression (Maddocks and Oyston, 2008). Transcriptional regulation by LTTRs can occur at the local level – i.e., of adjacent genes – or at the global level of genes located elsewhere along the chromosome (Heroven and Dersch, 2006; Hernandez-Lucas et al., 2008). In addition, LTTRs displaying positive or negative autoregulation have been described (Heroven and Dersch, 2006; Hernandez-Lucas et al., 2008). Consistent with the widespread presence of LTTRs, diverse external stimuli modulate their activity and the genes they regulate are involved in a wide range of functions such as CO_2_ fixation, amino acid biosynthesis, quorum sensing, cell cycle, exopolysaccharide biosynthesis and motility (Maddocks and Oyston, 2008). In addition, genes involved in secondary metabolite (SM) synthesis in *Burkholderia thailandensis* – a closely related model organism of *B. pseudomallei* – were shown to be regulated by a LTTR (Mao et al., 2017). Both *B. thailandensis* and *B. pseudomallei* have an extensive secondary metabolism. Previous studies reported that disruptions of single SM gene clusters, such as the malleilactone siderophore or the bactobolin cluster, for example, leads to a strong attenuation of virulence of the bacteria in mice and worms (Carr et al., 2011; Biggins et al., 2012, 2014; Amunts et al., 2015). Furthermore, several LTTRs have been shown to play a crucial role in host–pathogen interactions by controlling expression of virulence genes. The LTTR ShvR of *Burkholderia cenocepacia* for instance regulates transcription of protease and type 2 secretion system (T2SS) genes and disruption of *shvR* attenuates virulence in a mammalian and plant model of infection (O’Grady and Sokol, 2011; O’Grady et al., 2011; Subramoni et al., 2011). YtxR of *Yersinia enterocolitica* activates expression of an ADP-ribosyltransferase toxin while MexT of *Pseudomonas aeruginosa* was shown to repress T3S (Axler-Diperte et al., 2006; Tian et al., 2009). The LTTR MvfR of *P. aeruginosa* acts as global virulence regulator and is required for full virulence in mice and plants (Cao et al., 2001; Deziel et al., 2005).

*B. pseudomallei* is a facultative intracellular bacterium capable of escaping from the phagosome into the cytoplasm of the host cell and direct cell-to-cell spread, which is mediated by the T3SS-1 and the T6SS-1, respectively (Kespichayawattana et al., 2000; Burtnick et al., 2008, 2011; Chen et al., 2011; French et al., 2011). In the present study, we functionally analyzed a *B. pseudomallei* transposon mutant that was identified in a previously performed transposon mutagenesis screen (Pilatz et al., 2006). The mutant displayed reduced plaque formation on host cell monolayers indicating a defect in the intracellular life cycle, as plaque formation is attributed to cell-to-cell spread. The transposon of this mutant inserted into the previously uncharacterized gene BPSL0117, which is annotated as a LTTR. Using global transcriptome analysis we show that BPSL0117 is a pleiotropic global regulator of a wide variety of genes involved in iron and amino acid metabolism, SM, T3SS3 and T6SS1 and T6SS2. The BPSL0117 mutant was unable to proliferate in primary macrophages and was strongly attenuated in virulence to mice demonstrating a central role of the LTTR in the virulence of *B. pseudomallei*. We designated the novel LTTR GvmR for “globally acting virulence and metabolism regulator.”

## Materials and Methods

### Bacterial Strains, Media, Reagents, and Growth Conditions

*B. pseudomallei* E8 is a soil isolate from the area surrounding Ubon Ratchathani, north-east Thailand (Wuthiekanun et al., 1996). *B. pseudomallei* was grown on Columbia agar or Luria-Bertani (LB) agar plates and LB broth or M9 minimal broth containing 0.4% glucose as a carbon source was used as liquid media. When appropriate, antibiotics were added at the following concentrations: 25 μg ml^-1^ chloramphenicol, 25 and 12.5 μg ml^-1^ tetracycline for *E. coli* SM10(pOT182), 100 μg ml^-1^ streptomycin and 50 μg ml^-1^ tetracycline for *B. pseudomallei* Tn*5* (pOT182) mutants, 100 μg ml^-1^ ampicillin for *E. coli* DH5α (pTNS3) and 35 μg ml^-1^ kanamycin for *E. coli* HB101(pRK2013). All chemicals were obtained from Sigma-Aldrich unless stated otherwise. *B. pseudomallei* experiments were carried out in biosafety level 3 (BSL3) laboratories.

### *B. pseudomallei* Tn*5*-OT182 Mutagenesis and Plaque Assay Screening

Genome-wide mutagenesis of *B. pseudomallei* E8 was performed with Tn*5*-OT182 followed by an analysis of the mutants for their plaque forming ability using Ptk2 cells as previously described (Pilatz et al., 2006). Mutants that exhibited reduced plaque formation compared with the wild type strain were selected to determine the transposon insertion site as described previously (Pilatz et al., 2006). In one of the mutants, the transposon inserted behind base 277 of the BPSL0117 locus. This mutant was selected for further analysis and termed *B. pseudomallei* Δ*gvmR* (ΔBPSL0117).

### Complementation of *B. pseudomallei* Δ*gvmR*

To complement the *B. pseudomallei* Δ*gvmR* mutant we used the mini-Tn*7* system described by Choi et al. (2008). The primers 0117compF-*Hin*dIII (atAAGCTTGACGCTTTTTATCGCAA CTCTCTACTGTAGATGAGCGATGGAAGACGG) and 0117compR-*Kpn*I (atGGTACCTTGCTTTTGGCGTAGGAGAT) (restriction sites underlined) were used to amplify the *gvmR* coding region from *B. pseudomallei* E8. The forward primer contains a P_BAD_-promotor, which is constitutively active in *B. pseudomallei* (Qiu et al., 2008). Following digestion, the PCR product was ligated into the *Kpn*I/*Hin*dIII sites of the pUC18T mini-Tn*7*T-Zeo vector (Choi et al., 2008), cloned into *E. coli* DH5α, and delivered into *B. pseudomallei* ΔBPSL0117 by four parental mating using the donor strain *E. coli* DH5α pUC18T mini-Tn*7*T-Zeo-BPSL0117 and the *E. coli* helper strains *E. coli* Hb101 (pRK2013) and *E. coli* DH5α (pTNS3) (Choi et al., 2008). Briefly, bacteria were cultured with the appropriate antibiotics overnight in LB medium. One hundred microliters of each of the cultures was mixed with 600 μl 10 mM MgSO_4_ and centrifuged at 7000 *g* for 2 min. The supernatant was removed and the cells were washed with 1 ml of 10 mM MgSO_4_ and finally resuspended in 30 μl 10 mM MgSO_4_. Bacteria were spread on a 0.45 μm filter placed on prewarmed LB agar plates containing 4% (v/v) glycerol and incubated for 8 h at 37°C. Cells were then harvested, resuspended in 2 ml PBS and spread onto LB agar containing 4% (v/v) glycerol, 2 mg ml^-1^ zeocin, and 15 μg ml^-1^ polymyxin B to select for complemented mutants. Successful insertion of P_BAD_-BPSL0117 into one of the attTn*7* sites of the recipient strain *B. pseudomallei* Δ*gvmR* was verified by PCR using the primer TnL7 (ATTAGCTTACGACGCTACACCC) and one of the site specific primers BPGLMS1 (GAGGAGTGGGCGTCGATCAAC), BPGLMS2 (ACACGACGCAAGAGCGGAATC), and BPGLMS3 (CGGACAGGTTCGCGCCATGC). The generated complemented mutant was termed *B. pseudomallei* Δ*gvmR*::*gvmR*.

### Motility Assays

Bacteria were grown overnight in LB broth and adjusted to an OD_650__nm_ of 0.25 in sterile PBS. One microliter of the bacteria was spotted into LB soft agar plates. Swimming and swarming motility was determined using 0.3% (w/v) agar and 0.6% (w/v) agar, respectively. The plates were incubated at 37°C for 24 and 48 h after which the diameter of the circular expansion pattern of bacterial migration from the point of inoculation was measured at the indicated time points.

### Biofilm Assay

A volume of 0.5 ml LB in 5 ml polystyrene tubes was inoculated with overnight cultures of bacteria at an OD_650__nm_ of 0.01. Bacteria were grown statically at 37°C for 24 and 48 h. The medium was discarded, and the tubes were washed with sterile water. To stain adherent biofilms, 2 ml of 1% (w/v) crystal violet solution was added to each tube and incubated at room temperature (RT) for 15 min. The solution was then discarded and the tubes were washed three times with sterile water to remove unbound crystal violet. Finally, bound dye was eluted with 2 ml of 100% methanol, the absorbance of the solution was measured at 540 nm.

### RNA Extraction

For microarray experiments, total RNA was extracted from the *B. pseudomallei* E8 wild type and the Δ*gvmR* mutant grown in M9 minimal medium at the exponential growth phase (OD_650__nm_ of 0.5). Fifty milliliters of cell suspension were harvested and centrifuged at 2°C at 10,000 *g* for 5 min. The cell pellets were quickly resuspended in 1 ml of Trizol Reagent (Invitrogen). After the RNA extraction procedure according to manufacturer’s instructions, the integrity of the RNA was assessed by agarose gel electrophoresis and tested for the absence of DNA contamination by PCR.

### *B. pseudomallei* K96243 Tiling Microarrays and Expression Profiling

High-density tiling arrays were fabricated by Roche NimbleGen (Roche NimbleGen, United States) based on the *B. pseudomallei* K96243 reference genome (Holden et al., 2004). Three independent RNA preparations from each strain were reverse transcribed and Cy3 or Cy5 labeled. Briefly, to obtain DNA-free RNA, 100 μg total RNA per sample were treated with DNA-*free*^TM^ Kit from Ambion (United States) according to the manufacturer’s instructions. After precipitation of the RNA with ethanol and resuspension in nuclease free water, 10 μg per sample were subjected to ribosomal RNA depletion using the MICROB*Express*^TM^ kit from Ambion (United States) according to the manufacturer’s instructions. Afterward, 2 μg of purified mRNA were used for cDNA synthesis utilizing Superscript II Reverse Transcriptase (Life Technologies). Finally, 1.5 μg of obtained cDNA were used for the labeling procedure using the ULS^TM^ arrayCGH Labeling Kit (Leica Microsystems) according to the manufacturer’s instructions. Hybridization and microarray scanning were processed as described before (Nandi et al., 2010; Ooi et al., 2013). Microarray images were analyzed by Roche NimbleScan software (Roche NimbleGen, United States). All arrays were normalized using Robust Multi-array Average (RMA) (Roche NimbleGen, United States). Only those probes downstream of the translational start site were considered for estimating the fold change of gene expression. Ratios obtained for probes corresponding to the same gene were averaged and normalized signals from all three biological replicates were averaged to obtain a single value. Genes showing a ratio of log_2_ < -1 or log_2_ > 1 were considered as differentially expressed. Microarray data has been deposited in the Gene Expression Omnibus (GEO) under accession number GSE110883.

### Quantitative Real-Time PCR

For quantitative real-time PCR (qRT-PCR) analysis, *B. pseudomallei* wild type E8 and Δ*gvmR* mutant were grown in M9 minimal medium to exponential phase (OD_650__nm_ of 0.5). The cells were collected by centrifugation (8500 *g*, 2°C) for 5 min and suspended in 1 ml of Trizol Reagent (Invitrogen). Total RNA was extracted according to manufacturer’s instructions and RNA integrity was assessed by agarose gel electrophoresis. DNA was removed using DNase I (Thermo Fisher Scientific) and its absence was confirmed by PCR. Reverse transcription for qRT-PCR was performed using 5 μg of total RNA, 200 U of Superscript II Reverse Transcriptase (Invitrogen) and 500 ng of random primers, following the manufacturer’s instructions. Quantitative PCR amplification of the resulting cDNA was performed with Platinum SYBR Green (Applied Biosystems) and gene-specific primers (Supplementary Table S1). The primers were designed using the OligoPerfect primer designing tool from Invitrogen^1^. PCRs were run in triplicate. Results were normalized using the 23S rRNA gene displaying constant expression levels as endogenous control as previously described (Kumar et al., 2008). Relative expression levels were calculated using the 2^-ΔΔ*C*_T_^ method (Livak and Schmittgen, 2001).

### Infection of RAW264.7 Macrophages

RAW 264.7 cells were cultivated in Dulbecco’s Modified Eagle Medium (DMEM, Thermo Fisher Scientific, United States) supplemented with 10% fetal calf serum (FCS, Capricorn GmbH, Germany). Twenty-four hours prior to infection, cells were seeded in 48-well plates at 9 × 10^4^ cells per well. *B. pseudomallei* wild type, Δ*gvmR* mutant or the complemented mutant Δ*gvmR*::*gvmR* were added to the macrophages at MOI1 followed by low speed centrifugation at 120 *g* for 4 min at RT to initiate infection. After incubation at 37°C for 30 min, extracellular bacteria were removed by washing the cells twice with PBS and incubating the cells in fresh medium containing 250 μg ml^-1^ kanamycin (time 0; 0 h). Infected cells were incubated for 6 and 24 h to measure intracellular survival and replication. For this, the number of intracellular colony forming units (CFU) was determined per well by plating serial dilutions of the Tergitol(1%)-lysed cells on LB agar plates.

### Generation and Infection of Bone Marrow-Derived Macrophages

Murine bone marrow-derived macrophages (BMMs) were generated under serum-free conditions from BALB/c mice as previously described (Eske et al., 2009). Briefly, tibias and femurs were aseptically removed, and bone marrow cells were flushed out with sterile PBS and centrifuged at 150 *g* for 10 min. Cells were resuspended in RPMI medium containing 5% Panexin BMM (PAN Biotech), recombinant murine granulocyte-macrophage colony-stimulating factor (2 ng ml^-1^; PAN Biotech) and 50 μM mercaptoethanol and cultivated for at least 10 days at 37°C and 5% CO_2_. Twenty-four hours prior to infection, cells were seeded in 48-well plates at 1.5 × 10^5^ cells per well and infected with *B. pseudomallei* wild type, Δ*gvmR* or the complemented mutant Δ*gvmR*::*gvmR* at MOI2 as described above. After 30 min of incubation, extracellular bacteria were removed by washing twice with PBS and incubation in fresh medium supplemented with 250 μg ml^-1^ kanamycin (time 0; 0 h). At the indicated time points the number of intracellular CFU was determined as described above.

### Murine Infection Model

Female 8- to 12-week-old BALB/c mice were obtained from Charles River Wiga Deutschland GmbH (Sulzfeld, Germany). All *in vivo* studies were approved by the local authority. Animals were maintained under specific pathogen-free conditions and were provided with food and water *ad libitum*. Bacteria were grown for 16 h on LB agar supplemented with 5% sheep blood and adjusted to an OD_650__nm_ of 0.25 in sterile PBS. Prior to intranasal (*i.n*.) application mice were anesthetized with a mixture of ketamine hydrochloride and xylazine hydrochloride. Thirty microliters of the bacterial suspension were inoculated into both nostrils of an animal (15 μl in each nostril). Animals were monitored daily for signs of disease and mortality. To enumerate bacteria in the spleen, liver, and lungs, the organs were aseptically removed 48 h after infection, homogenized in 0.5–1 ml sterile PBS containing 0.5% Tergitol and 1% BSA. The suspensions were diluted and plated on LB agar plates to determine the number of CFUs per organ. All animal studies were conducted under a protocol approved by the Landwirtschaft, Lebensmittelsicherheit und Fischerei Mecklenburg-Vorpommern (LALLF M-V; 7221.3-1.1-020/11). All efforts were made to minimize suffering and ensure the highest ethical and humane standards.

### Statistical Analysis

Data are expressed as mean values ± standard error of the mean (SEM) and analyzed using one-way ANOVA with Bonferroni multiple comparisons posttest as indicated in the figure legends. Gene expression data obtained by DNA microarray experiments were analyzed using Student’s *t*-test. Survival data of the mice were analyzed using the Kaplan–Meier method. A *p*-value of <0.05 was considered statistically significant.

## Results

### Insertion of the Tn*5* Transposon Into the BPSL0117 Open Reading Frame Leads to Decreased Plaque Formation

**FIGURE 1:**
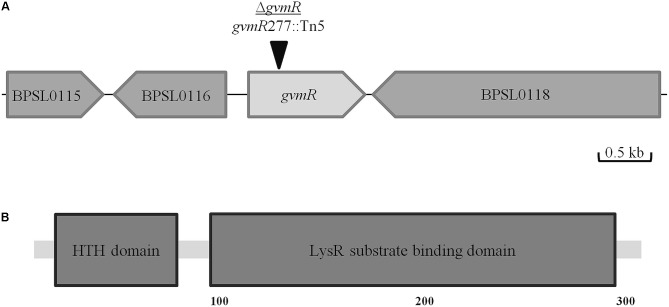
Localization of the Tn*5* insertion site in the Δ*gvmR* mutant. **(A)** Schematic representation of the Tn*5* insertion site in BPSL0117 (*gvmR*) after nucleotide 277 (black triangle). Annotation of the neighboring genes: BPSL0115, patatin-like phospholipase; BPSL0116, D-3-phosphoglycerate dehydrogenase/2-oxoglutarate reductase; BPSL0118, DNA topoisomerase. **(B)** Domain organization of GvmR predicted by Simple Modular Architecture Research Tool (SMART, http://smart.embl-heidelberg.de/). HTH, helix-turn-helix DNA binding domain (PFAM00126); LysR substrate binding (PFAM03466).

We previously performed a genome-wide transposon mutagenesis of *B. pseudomallei* E8 to identify novel gene products involved in the intracellular life cycle of the bacteria (Pilatz et al., 2006). For this, the ability of the mutants to spread intercellularly was analyzed using the plaque formation assay and Ptk2 cells. In the present study, one of the transposon mutants that showed markedly reduced plaque formation as compared with the wild type was selected for functional characterization. In this mutant, the Tn*5* transposon inserted into the hitherto uncharacterized ORF AP949_RS18685 (*B. pseudomallei* strain E8)^2^, which is a homolog of BPSL0117 of the *B. pseudomallei* reference strain K96243 (100% protein sequence identity). The ORF is located on chromosome I and flanked by two genes annotated as a D-isomer specific 2-hydroxyacid dehydrogenase and DNA topoisomerase III (**Figure 1A**). AP949_RS18685 is annotated as a LysR family transcriptional regulator of 351 amino acids in length. Using the Simple Modular Architecture Research Tool (SMART^3^), we found that the protein contains a LysR substrate binding domain (e-value: 8.2e-49; coverage: 87–294 aa) and an N-terminal HTH domain (e-value: 1.3e-18; coverage: 4–63 aa) (**Figure 1B**). The protein secondary structure program Jpred^4^ supports the presence of a HTH domain and showed that it is connected with the downstream substrate binding domain by a helical linker. Such domain architectures have been already described for other LTTRs (Maddocks and Oyston, 2008). Interestingly, global expression analysis of *B. pseudomallei* showed that BPSL0117 (AP949_RS18685) is significantly upregulated during acute and chronic infection of hamsters and rats, respectively (Tuanyok et al., 2006; van Schaik et al., 2008). We have named BPSL0117 GvmR (for globally acting virulence and metabolism regulator). For the functional analysis of the *B. pseudomallei* mutant harboring the Tn*5* transposon in the *gvmR* gene, disruption of *gvmR* was complemented at a neutral site in the chromosome using the mini-Tn7 system (Δ*gvmR*::*gvmR*) (Choi et al., 2008).

### GvmR Contributes to Survival and Intracellular Replication of *B. pseudomallei* in Phagocytes

**FIGURE 2:**
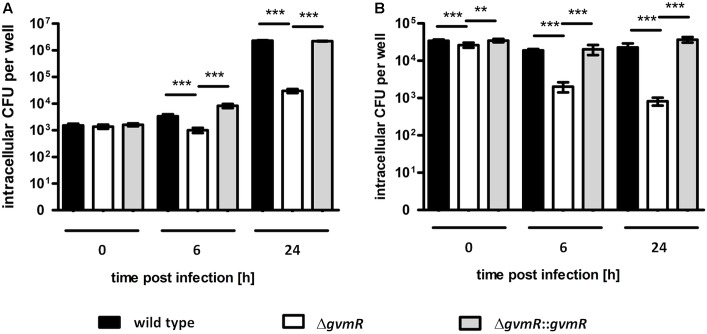
Uptake and intracellular replication of *B. pseudomallei* E8 wild type, Δ*gvmR* mutant and complemented mutant Δ*gvmR*::*gvmR* in **(A)** RAW 264.7 macrophages and **(B)** BALB/c-BMM macrophages. The bacteria were grown on LB agar supplemented with 5% sheep blood for 16 h at 37°C and diluted in PBS for the infection. RAW264.7 macrophages and BALB/c-BMM macrophages were infected at an MOI of 1 and 2, respectively, and intracellular CFUs were determined using the antibiotic protection assay followed by plating of infected cells on LB agar. 0 h indicates bacterial uptake and intracellular bacterial survival and replication was quantified at 6 and 24 h postinfection. Shown are mean values of three independent experiments performed in triplicate. Error bars indicate standard error of the mean (SEM). Statistical analyses were performed using one-way ANOVA with Bonferroni multiple comparisons posttest (^∗∗^*p* < 0.01; ^∗∗∗^*p* < 0.001).

Given the reduced ability of the Δ*gvmR* mutant to form plaques on cell monolayers, which indicates a defect in one of the stages of the intracellular life cycle, we examined the survival and replication of the mutant inside phagocytic host cells. To this end, phagocytic cells were selected, i.e., the murine RAW 264.7 macrophage cell line and primary bone marrow-derived macrophages obtained from BALB/c mice (BALB/c-BMM). The cells were infected with *B. pseudomallei* wild type, Δ*gvmR* mutant and complemented mutant followed by an antibiotic protection assay. To quantify intracellular survival and replication, the number of intracellular bacteria was determined at a very early time point postinfection (0 h) and at later time points (6 and 24 h), respectively. Entry into RAW 264.7 macrophages was similar between the strains indicating that the inactivation of *gvmR* did not influence this process (**Figure 2A**). In addition, the Δ*gvmR* mutant was able to survive and proliferate inside RAW 264.7 macrophages as indicated by a 25-fold increase in CFU at 24 h postinfection relative to 0 h. However, the number of intracellular Δ*gvmR* mutant bacteria was considerably lower compared with wild type and complemented mutant at 6 and 24 h postinfection (**Figure 2A**). This result shows that in the absence of a functional GvmR growth of *B. pseudomallei* inside RAW 264.7 macrophages is impaired. Both the wild type and complemented mutant exhibited intracellular CFUs that remained similar over the course of the infection of primary BMMs (**Figure 2B**). This finding suggested that the strains were able to survive but were incapable of robust replication inside BMM. In contrast, the Δ*gvmR* mutant displayed a gradual decrease in intracellular CFUs at 6 h and 24 h postinfection in comparison with the 0 h time point by 14- and 39-fold, respectively. The decline in the number of intracellular Δ*gvmR* mutant bacteria indicates a reduced ability of the bacteria to survive intracellularly and to resist killing by BMMs. Importantly, complementation of *gvmR* (Δ*gvmR*::*gvmR*) restored the full wild type phenotype indicating that the insertion of Tn*5* into the gene did not produce polar effects. In summary, the data demonstrate that GvmR plays a vital role in the survival and growth of *B. pseudomallei* in phagocytic cells.

### The Δ*gvmR* Mutant Is Severely Attenuated in Virulence to Mice

**FIGURE 3:**
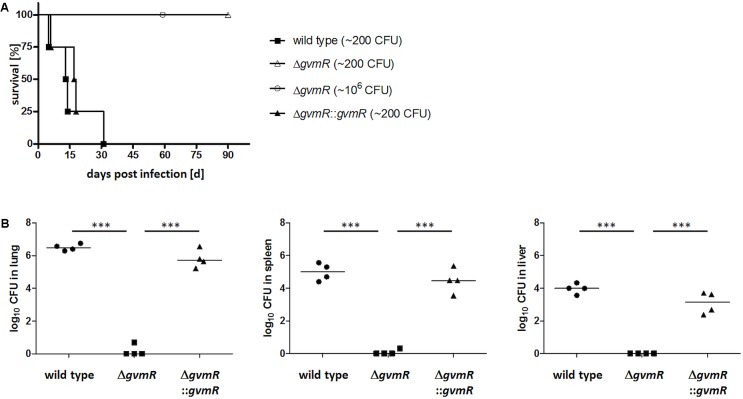
Survival curves of BALB/c mice **(A)** and bacterial loads **(B)** in lung, spleen, and liver after infection with *B. pseudomallei* E8 wild type, Δ*gvmR* mutant, and complemented mutant Δ*gvmR*::*gvmR*. **(A)** Mice (*n* = 4) were infected intranasally with a low dose (∼200 CFU) of all strains. In addition, mice were inoculated intranasally with a high dose (∼10^6^ CFU) of the Δ*gvmR* mutant. Data from two independent experiments are shown. Curves were compared by using the log-rank Kaplan–Meier test (*p* = 0.0001). **(B)** Bacterial loads in lung, spleen, and liver were determined 48 h postinfection with ∼300 CFU of *B. pseudomallei* wild type, Δ*gvmR* mutant and complemented strain Δ*gvmR*::*gvmR*. Each symbol represents the CFU from one organ. The horizontal lines represent the geometric means. Statistical analyses were performed by using one-way ANOVA with Bonferroni multiple comparisons posttest (^∗∗∗^*p* < 0.0001).

To examine whether GvmR contributes to *in vivo* virulence, an intranasal model of infection in mice was used. BALB/c mice were inoculated intranasally with approximately ∼200 CFU of the *B. pseudomallei* wild type, Δ*gvmR* mutant and complemented mutant and monitored for survival for 90 days after infection. Following challenge with wild type bacteria, all mice succumbed to the infection within 30 days postinfection (**Figure 3A**). By contrast, all mice survived the infection by Δ*gvmR* mutant. Likewise, a 10,000-fold increase (∼10^6^ CFU) in the infectious dose resulted in 100% survival demonstrating a severe attenuation of the Δ*gvmR* mutant. Complementation of the *gvmR* disruption restored the ability of *B. pseudomallei* to cause lethal infections within 30 days postinfection (**Figure 3A**). To analyze whether the virulence loss of the mutant was due to its impaired survival and/or dissemination in mice, we determined the bacterial loads in lung, spleen, and liver 48 h postinfection. While high numbers of wild type and complemented mutant bacteria were detected in all three organs exceeding the CFU used for inoculation of the mice, almost no Δ*gvmR* mutant bacteria were recovered from the lung, spleen, and liver (**Figure 3B**). These data show that the GvmR regulator is required for survival and virulence of *B. pseudomallei in vivo*.

### Disruption of *gvmR* Impairs Growth in Minimal Medium, Biofilm Formation, and Motility

**FIGURE 4:**
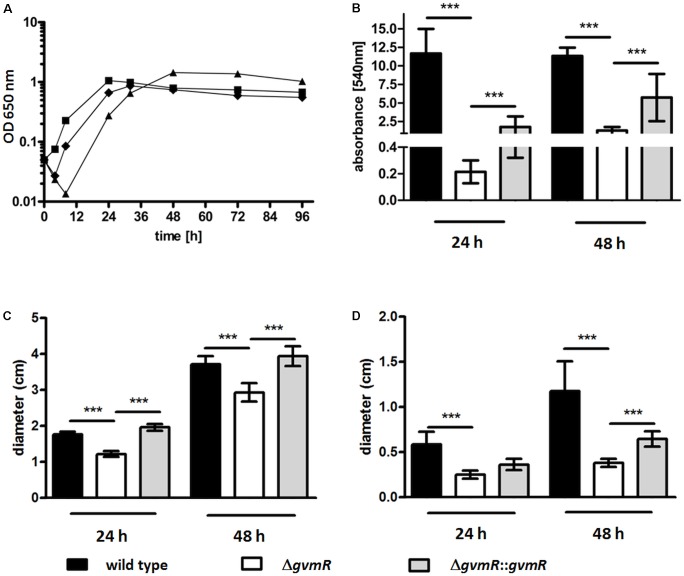
Growth **(A)**, biofilm formation **(B)**, and motility **(C,D)** of *B. pseudomallei* E8 wild type, Δ*gvmR* mutant and complemented mutant Δ*gvmR*::*gvmR*. **(A)** Bacteria were grown in M9 minimal medium at 37°C and 140 rpm for 96 h. Shown are mean values of two independent experiments. Error bars indicate standard error of the mean (SEM). black square – wild type, black triangle – Δ*gvmR*, black diamond – complemented strain Δ*gvmR*::*gvmR*. **(B)** Biofilm formation was quantified 24 and 48 h after static incubation in LB broth at 37°C. Crystal violet retention of the biofilm mass was measured at 540 nm after methanol elution of the dye. Data represent mean values of three experiments. Error bars indicate standard error of the mean (SEM). Statistical analyses were performed by using one-way ANOVA with Bonferroni multiple comparisons posttest (^∗∗∗^*p* < 0.001). **(C)** Swimming motility of the indicated strains in LB agar (0.3%) and **(D)** swarming motility in LB agar (0.6%). Swimming and swarming motility was quantified 24 and 48 h after incubation at 37°C by measuring the diameters of the circular bacterial migration from the point of inoculation. Shown are mean values of three independent experiments. Error bars indicate standard error of the mean (SEM). Statistical analyses were performed using one-way ANOVA with Bonferroni multiple comparisons posttest (^∗∗∗^*p* < 0.001).

Inactivation of *gvmR* did not affect growth of the bacteria in LB medium (Supplementary Figure S1). However, in M9 minimal medium using glucose as the sole carbon source, the lag phase of the Δ*gvmR* mutant was characterized by a net decrease in bacterial cell numbers delaying the transition into exponential phase compared with the wild type (**Figure 4A**). OD_650__nm_ values of the Δ*gvmR* mutant were similar to those of the wild type and the complemented mutant at early stationary phase and slightly higher at later stages of this growth phase. Next, the biofilm forming capacity of the Δ*gvmR* mutant was investigated under static conditions in LB medium using crystal violet staining. Disruption of *gvmR* severely reduced the ability of *B. pseudomallei* to form biofilms after 24 h and 48 h incubation, which could be partially restored by complementation (**Figure 4B**). Furthermore, the Δ*gvmR* mutant displayed significantly reduced swimming motility in LB agar in comparison with the wild type and complemented mutant at 24 and 48 h (**Figure 4C**). Likewise, the swarming motility of the Δ*gvmR* mutant in LB agar was significantly lower relative to the wild type at 24 h and significantly lower compared with the wild type and complemented mutant at 48 h (**Figure 4D**). Altogether, the data show that GvmR is involved in the regulation of growth under nutrient-limited conditions, biofilm formation, and flagellum-based motility.

### Transcriptome Alterations in *B. pseudomallei* Δ*gvmR*

The results described above show that the Δ*gvmR* mutant exhibited reduced growth in M9 minimal medium but not in LB (**Figure 4A** and Supplementary Figure S1). This finding indicates that GvmR is necessary for proliferation of the bacteria specifically under nutrient limited conditions. Thus, to identify genes regulated by GvmR, gene expression profiles of *B. pseudomallei* wild type and Δ*gvmR* mutant grown in M9 minimal media to exponential phase were compared using whole genome high density tiling arrays (Roche). In addition, 27 differentially regulated genes representing different functional categories were selected for verification by qRT-PCR. For the sake of usability of the data, we used the gene locus ID nomenclature of the reference strain K96243.

**FIGURE 5:**
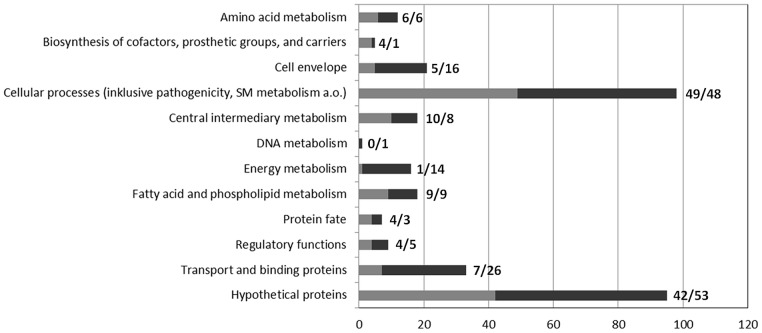
Functional classification of genes differentially expressed in the Δ*gvmR* mutant. Genes that were up- and downregulated in the Δ*gvmR* mutant as compared with wild type are indicated in black and gray, respectively. The number of genes per functional category is shown at the bottom. The number of down- and upregulated genes per functional category are shown next to the bars (down/up).

As revealed by transcriptomic analysis, a total of 331 genes were differentially expressed in the Δ*gvmR* mutant versus the wild type (log_2_ < -1 or >1, *p* < 0.05; Supplementary Table S2). A total of 141 genes were found to be downregulated and 190 genes were upregulated indicating that GvmR acts as both an activator and repressor of transcription. The majority of these genes (71.4%) encodes proteins with known or annotated function. Functional annotation was validated on the basis of sequence similarity and domain analysis and genes were grouped into functional categories according KEGG^5^. Genes that were regulated by GvmR belong to diverse functional categories indicating that the regulator has a broad influence on the transcriptome of *B. pseudomallei* (**Figure 5**).

### Amino Acid, Carbon, and Energy Metabolism

The microarray data showed that transcript levels of 12 genes involved in amino acid metabolism were differentially modulated in the Δ*gvmR* mutant. Genes such as BPSL1691 (*metZ*), BPSL0197 (*metX*) (cysteine and methionine metabolism), BPSS0005 (*kbl*) and BPSS0006 (*tdh*) (glycine, serine, and threonine metabolism) were found to be downregulated in the mutant while others were upregulated, e.g., BPSL2545 (*metE*) (cysteine and methionine metabolism), BPSL2738 and BPSL2739 (*hmgA*) (tyrosine metabolism). A gene cluster participating in branched-chain amino acid transport (BPSL3416-3418) displayed increased expression in the absence of a functional *gvmR* gene. Furthermore, disruption of *gvmR* altered transcription of several genes related to energy and carbon metabolism. The complete F0F1 ATPase gene cluster (BPSS1945-1953) as well as the *adhA* (BPSS1944; alcohol dehydrogenase) located immediately downstream were upregulated in the Δ*gvmR* mutant. Interestingly, the expression of the F0F1 ATPase-encoding gene cluster located on chromosome 1 (BPSL3394-3403) was not significantly affected by GvmR inactivation. Likewise, the 6-phosphofructokinase (BPSS1957), the acetate kinase (BPSS1956), the phosphate acetyltransferase (BPSS1955), and the phosphoenolpyruvate phosphomutase (BPSS0610) involved in glycolysis, gluconeogenesis, phosphonate, or acetate metabolism showed increased transcript levels in the mutant. Upregulation of the 2-methylisocitrate lyase gene (*prpB*; BPSS0206) and simultaneous downregulation of the isocitrate lyase gene (*aceA*; BPSL2188) was observed in Δ*gvmR*, which suggests a disturbance of the anaplerotic function of the TCA cycle (Munoz-Elias and McKinney, 2005, 2006). qRT-PCR confirmed the differential regulation of the selected genes *kbl*, BPSS1955, *aceA*, and *prpB* (Supplementary Figure S2A). Altogether, the dysregulation of diverse metabolic pathways may explain growth retardation of the Δ*gvmR* mutant under nutrient-limited conditions.

### Motility, Transport, and Stress Adaptation

Our analysis revealed a differential regulation of 17 flagella-motility associated genes, the majority of which was upregulated in the Δ*gvmR* mutant. The BPSL3310-3311 regulator was one of the most upregulated genes in the Δ*gvmR* mutant (log_2_ ratio: -1.75 to 2.39). However, transcription of a flagella gene cluster comprising BPSL3319 (*fliC*; flagellin); BPSL3320 (*fliD*; flagellar hook-associated protein); and BPSL3321 (flagella protein) was found to be reduced (log_2_ ratio: 1.06–1.61). Two porins were among the most strongly regulated genes: the expression of BPSL3036 was strongly increased upon disruption of *gvmR* (log_2_ ratio: -3.13) and BPSS0879 was the most downregulated gene in the Δ*gvmR* mutant (log_2_ ratio: 4.47). Both results were confirmed by qRT PCR (Supplementary Figure S2B). The substrates of these porins are currently unknown. Furthermore, elevated transcript levels of universal stress proteins (BPSS0836-0839), chaperones (BPSL1323, BPSS2288), and peroxiredoxins (BPSL0302 and BPSL3019) were detected in the Δ*gvmR* mutant suggesting that inactivation of the regulator imposes stress on the bacteria when nutrient availability is limited.

### Iron Acquisition and Iron-Sulfur (Fe-S) Incorporation

The availability of iron is of particular importance for pathogenic bacteria and hence iron acquisition plays a crucial role in host–pathogen interactions. Disrupting *gvmR* induced a gene cluster comprising the iron permease gene (BPSS1999) and cupredoxin-like domain containing gene (BPSS2000) (log_2_ ratio: -1.8 and -1.9, respectively). qRT-PCR of BPSS2000 verified the values obtained by tiling arrays (Supplementary Figure S2A). Similarly, the complete gene cluster encoding an iron-hydroxamate ABC transport system including an ECF sigma-70 factor (BPSL1781-1787) displayed higher expression levels in the mutant than in the wild type. Increased expression of BPSL1787 in the mutant was confirmed by qRT-PCR (Supplementary Figure S2A). Transcription of six genes belonging to the Fe-S cluster (ISC) assembly machinery cluster BPSL2290-2283 were reduced in the absence of a functional GvmR and qRT PCR of BPSL2289 verified this result (Supplementary Figure S2A). The ISC cluster is believed to be important for Fe-S biogenesis in the absence of stress conditions. *B. pseudomallei* harbors a second Fe-S cluster biosynthetic system (Suf; BPSL2369-BPSL2374), which is thought to be specifically active under iron limitation (Ayala-Castro et al., 2008). Although induction of the iron transport related genes described above might indicate iron deficiency, expression levels of the Suf gene cluster were similar between wild type and mutant.

### Secondary Metabolism

The genomes of *B. pseudomallei* and *B. thailandensis* are enriched in biosynthetic gene clusters involved in secondary metabolism. Experimental characterization of some of the polyketide synthase (PKS) and non-ribosomal peptide synthetase (NRPS) gene clusters revealed a role in microbial competition and virulence (Duerkop et al., 2009; Biggins et al., 2014). We identified four SM gene clusters (SM cluster 1, 8, 12, 14, nomenclature from Biggins et al., 2014) that were differentially regulated by inactivating GvmR. Transcript levels of the SM gene cluster 14 (BPSS1266-1274) were significantly lower in the Δ*gvmR* mutant than in the wild type indicating its activation by GvmR (log_2_ ratio: 1.04–1.86). The metabolite synthesized by this cluster belongs to the family of syrbactins, which exert toxicity by inhibiting the proteasome (Biggins et al., 2014). Moreover, increased expression of the SM gene cluster 1 (BPSL1774-1778, NRPS/predicted metabolite: malleobactin), cluster 8 (BPSS0311-0299, PKS/metabolite: malleilactone), and cluster 12 (BPSS1174-1166, NRPS/PKS/metabolite: bactobolin) was observed in the Δ*gvmR* mutant. Bactobolin is an antibiotic while malleobactin and malleilactone are siderophores (Biggins et al., 2014; Amunts et al., 2015). qRT PCR experiments of selected genes supported the array data (Supplementary Figure S2C). In summary, our data show that GvmR is involved in the control of SM synthesis in *B. pseudomallei*.

### T3SS and T6SS Gene Clusters

*B. pseudomallei* harbors three T3SSs of which the animal pathogen like T3SS3 serves an important role in the intracellular life cycle and *in vivo* virulence of the bacteria (Stevens et al., 2004; Burtnick et al., 2008; Chen et al., 2014; Gutierrez et al., 2015; Willcocks et al., 2016; Vander Broek and Stevens, 2017). The T3SS3 gene cluster comprises 37 genes including several regulators and transposases (BPSS1516-1552). A total of 14 genes of the T3SS3 gene cluster were detected whose expression levels were significantly lower in the Δ*gvmR* mutant suggesting that GvmR induces expression of these genes (range log_2_ ratio: 1.13–2.17). Among the genes were for example *bipD* (BPSS1529; T3SS3 translocon protein/IpaD family), *bopA* (BPSS1524; putative T3SS3 effector protein), and *bicA* (BPSS1533; T3SS3 chaperone protein with regulatory function), which showed a 4.5-fold decrease. The majority of *bsa* genes located upstream of BPSS1533 and encoding predominantly T3SS3 apparatus proteins were not differentially regulated except for *bsaL* (BPSS1548; T3SS3 needle protein). Two genes coding for regulators were downregulated in the Δ*gvmR* mutant: *bprA* (BPSS1530; HNS-like regulatory protein) and *bprB* (BPSS1522; two component response regulator). qRT PCR confirmed the array results for four selected genes (*bprB, bopA, bipD*, and *bicA*) (Supplementary Figure S2D).

*B. pseudomallei* contain six T6SSs and homologs of five of them are present in *B. thailandensis*. The T6SS1 (also named T6SS-5) is an essential virulence factor in both bacteria (Burtnick et al., 2008; Schwarz et al., 2014; Si et al., 2017). The function of two other T6SSs has been investigated in *B. thailandensis*. The T6SS-6 (also named T6SS1) participates in interbacterial competition and the T6SS2 (also named T6SS-4) facilitates manganese uptake thereby influencing interbacterial interactions and virulence (Schwarz et al., 2010; Si et al., 2017). Our data show that the T6SS2 (BPSS0515-0534) and T6SS1 (BPSS1496-1511) are regulated by GvmR as expression levels of 11 T6SS2 genes and five T6SS1 genes were significantly lower in the Δ*gvmR* mutant than in the wild type, which was confirmed for BPSS1496 (*tssB*), BPSS1498 (*hcp*), and BPSS0517 by qRT PCR (Supplementary Figure S2D). The majority of the differentially regulated T6SS2 genes encode essential structural components of the secretion apparatus such as *hcp-2* (BPSS0518), *tssA-2* (BPSS0515), and *tssE-2* (BPSS0519). Similarly, all five of the T6SS-1 genes that were downregulated in the Δ*gvmR* mutant code for apparatus proteins: *tssB* (BPSS1496) and *tssC* (BPSS1497), *hcp* (BPSS1498), *tssE* (BPSS1499) and *tssF* (BPSS1500). *hcp* is the second most downregulated gene in the Δ*gvmR* mutant (log_2_ ratio: 3.11). A strong downregulation of *hcp* was also observed by qRT-PCR (Supplementary Figure S2D). Altogether, the GvmR-dependent upregulation of the T3SS3, T6SS2, and T6SS1 is consistent with and potentially explains the deficiency of the Δ*gvmR* mutant to multiply in BMMs and to cause lethal infections in mice (**Figures 2**, **3**).

### Genes of Unknown Function and Genes Unique to *B. pseudomallei*

Inactivation of GvmR increased expression of four gene clusters of unknown function that are unique to *B. pseudomallei* or to the Burkholderia genus (BPSL0937-0936, BPSL1618-1614, BPSL2743-2738, and BPSL2974-2973). Another gene cluster of unknown function comprising 21 genes (BPSL0493-0473) displayed reduced expression in the Δ*gvmR* mutant. Several of these ORFs showed similarity to citrate, fatty acid, or acyl-CoA metabolism-associated genes. However, the function of this cluster remains unknown. Interestingly, it exhibits a high degree of synteny and sequence similarity with the *afc* region found in bacteria belonging to the *Burkholderia cepacia* complex (Supplementary Figure S3 and Supplementary Table S3). The *afc* region controls production of the membrane-associated antifungal lipopeptide AFC-BC11 and is involved in biofilm formation and colony morphotypes by altering the composition of cell membranes (Kang et al., 1998; Subramoni et al., 2011, 2013). Further, a moderate downregulation of a cluster of genes (BPSS1639-1636) with high similarity to genes involved in itaconate degradation was detected in the Δ*gvmR* mutant. Itaconate is a mammalian compound that inhibits the bacterial isocitrate lyase, which is one of the essential enzymes of the glyoxylate shunt. This pathway facilitates survival of many bacteria during infection as well as under nutrient limited conditions (Sasikaran et al., 2014). qRT PCR results of one gene per operon confirmed that BPSL2974 and BPSL2743 were highly induced in the Δ*gvmR* mutant. However, the BPSL1638 gene located within the putative itaconate metabolism cluster showed a fold change of 0.54 (log_2_ = -0.907), which was below the threshold of log_2_ < -1 (Supplementary Figure S2B). Taken together, the data show that GvmR modulates the expression of a diverse set of genes of unknown function, which might play a role in growth under nutrient limitation or pathogen–host interaction.

## Discussion

**FIGURE 6:**
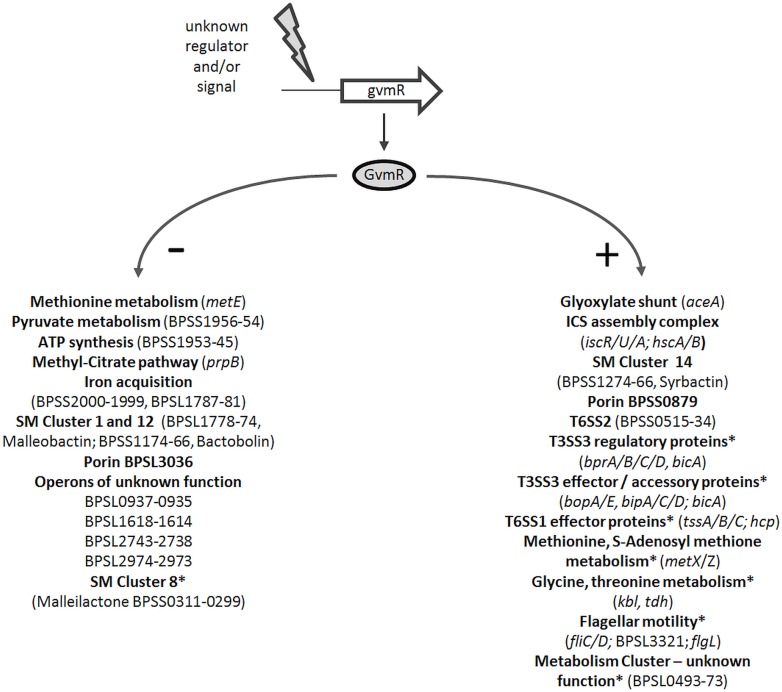
Schematic model depicting the regulatory activity of GvmR in *B. pseudomallei*. Shown are selected pathways and genes that are down- (-) and upregulated (+) by GvmR, which is activated by an as yet unknown signal or regulator. SM, secondary metabolite; ICS, iron-sulfur cluster assembly complex; ^∗^, regulation by BsaN/BicA; SM gene cluster numbering refers to Biggins et al. (2014).

Despite the implication of LTTRs in bacterial virulence and their high abundance in the genome of *B. pseudomallei* little is known about the identity of genes they regulate in these bacteria. This study is based on a functional analysis of a *B. pseudomallei* mutant, harboring a transposon in a predicted LTTR gene and displaying impaired intracellular growth. Using genome-wide transcriptome analysis and *in vitro* and *in vivo* infection assays we showed that GvmR modulates various cellular functions (**Figure 6**) and is necessary for virulence in mice. The findings indicate that GvmR functions as a global nutritional and virulence regulator, which is required to coordinate a cellular response that allows growth under nutrient limited conditions and in the animal host. A total of 331 genes were differentially expressed in the Δ*gvmR* mutant. Among these genes were several transcriptional regulators indicating that the identified GvmR regulon is composed of indirectly regulated genes. The genes that are directly controlled by GvmR remain to be determined in future studies. Consistent with its function as a global regulator, transcription of the *gvmR* neighboring genes was not altered in the Δ*gvmR* mutant.

Impaired growth in minimal medium suggests that GvmR is required to mount a transcriptional response for robust growth when nutrients are limited. Upregulation of genes involved in, for example, cysteine metabolism, fermentation (alcohol dehydrogenase), glycolysis, TCA cycle, and simultaneous downregulation of genes associated with amino acid metabolism (cysteine, methionine, and threonine) and the glyoxylate cycle (isocitrate lyase) in the Δ*gvmR* mutant relative to the wild type indicates a metabolic disturbance. The isocitrate lyase is an enzyme of the anaplerotic glyoxylate cycle, which allows growth on simple carbon sources such as fatty acids or acetate (Gould et al., 2006; Munoz-Elias and McKinney, 2006). This is particularly interesting as fatty acids are an important carbon source for bacteria during infection (Krivan et al., 1992; Campbell et al., 2003; Fang et al., 2005; Munoz-Elias and McKinney, 2005, 2006). Moreover, the isocitrate lyase is a critical factor for several intracellular pathogens including *B. pseudomallei* to persist during chronic infection (McKinney et al., 2000; Fang et al., 2005; van Schaik et al., 2009). Thus, the central metabolic pathways regulated by GvmR might be critical for growth and persistence in the animal host which is supported by the presented *in vivo* data. In fact, a previous transposon mutagenesis screen has identified genes associated with central metabolic functions such as histidine and purine biosynthesis that are necessary for intracellular proliferation of *B. pseudomallei* (Pilatz et al., 2006).

Furthermore, our data support the association of LTTRs with SM production, which has also been described in other bacteria previously. We identified GvmR as a positive and negative regulator of secondary metabolism (**Figure 6**). GvmR activated transcription of a gene cluster encoding a metabolite that belongs to the class of syrbactins, which induce cell death in mammalian, insect, and plant cells by proteasome inhibition (Clerc et al., 2009; Archer et al., 2010, 2012; Biggins et al., 2014; Dudler, 2014). Previous work showed that disruption of syrbactin synthesis caused a loss of virulence of *B. pseudomallei* in mice (Biggins et al., 2014). This is in agreement with our results that show an attenuated virulence of the Δ*gvmR* mutant. Furthermore, transcript levels of three other gene clusters involved in the synthesis of the siderophores malleilactone and malleobactin and the antibiotic bactobolin, respectively, were increased in the Δ*gvmR* mutant indicating that GvmR also acts as a negative regulator of SM production (Yang et al., 1993; Alice et al., 2006; Seyedsayamdost et al., 2010; Chandler et al., 2012; Kvitko et al., 2012). LTTRs were shown to stimulate the synthesis of the antibacterial compounds pyoluteorin and andrimid by *Pseudomonas* and *Serratia* species, respectively, or found to be located within biosynthetic gene clusters (Brodhagen et al., 2004; Li et al., 2012; Matilla et al., 2016). Noteworthy, recent work identified a quorum sensing regulated LTTR in *B. thailandensis* (ortholog in *B. pseudomallei*: BPSL2733), which up- or downregulates 13 of the 20 predicted biosynthetic gene clusters (Mao et al., 2017). While the overall regulon of this regulator is distinct from that of GvmR, both were involved in malleilactone and bactobolin gene expression. Discovery of novel bioactive compounds in *B. pseudomallei* and other microbes is hampered by the fact that biosynthetic gene clusters are often silent under standard laboratory conditions. Activating or deleting LTTRs might provide a widely applicable means of inducing silent gene clusters to uncover the diversity of SMs.

The microarray and qRT-PCR data show that GvmR is a positive regulator of key virulence factors of *B. pseudomallei*. Among the most strongly downregulated genes in the Δ*gvmR* mutant were genes encoding components of the T3SS3 and the T6SS1 apparatus. The T3SS3 and T6SS1 are essential for the intracellular life cycle of *B. pseudomallei* by mediating bacterial escape from the phagosome into the cytoplasm and intercellular spread via the formation of multinucleated giant cells, respectively (Burtnick et al., 2008; Chen et al., 2011, 2014; Gutierrez et al., 2015; Willcocks et al., 2016; Vander Broek and Stevens, 2017). Thus, decreased expression of T3SS3 and T6SS1 genes in the Δ*gvmR* mutant might enhance phagolysosomal killing and impair cell-to-cell spread leading to an inability of the Δ*gvmR* mutant to survive in primary macrophages as well as in mice. Consistent with this hypothesis, *B. pseudomallei* mutants harboring a disrupted T3SS3 or T6SS1 were previously shown to be attenuated for virulence *in vivo* (Stevens et al., 2004; Burtnick et al., 2011). Previous studies led to the identification of a regulatory cascade consisting of BspR (BPSL1105), BprP (BPSS1553), and BsaN (BPSS1546) that controls T3SS3 and T6SS1 gene expression (Chen et al., 2011, 2014). Transcript levels of the first two regulators were not affected in the Δ*gvmR* mutant indicating that GvmR does not influence T3SS3 and T6SS1 gene expression by acting on *bspR* and *bprB*. While *bsaN* transcription was not modulated by GvmR either, the chaperone gene *bicA* (BPSS1533) located in the T3SS3 gene cluster was downregulated in the mutant. BsaN requires BicA to directly regulate T3SS3 and T6SS1 gene expression, GvmR might indirectly influence transcription levels of the secretion system genes via *bicA* repression (Chen et al., 2014). However, expression of the two-component system VirAG (BPSS1494 and BPSS1495), which was previously shown to be activated by BsaN was not affected by GvmR mutation (Liu and Cheng, 2014). VirAG is located within the T6SS1 gene cluster and stimulates T6SS1 gene expression inside the host cell in response to glutathione (Lim et al., 2015). Likewise, the T3SS3 is strongly upregulated inside the host cell (Chen et al., 2011). However, the detection of significantly lower expression levels of T3SS3 and T6SS1 genes in the Δ*gvmR* mutant in minimal medium shows that the genes are also transcribed outside of host cells. This finding is consistent with previous transcriptome analysis in *B. pseudomallei* (Chen et al., 2014). Furthermore, we identified GvmR as a novel regulator of the T6SS2 and show that it is co-regulated with the T3SS3 and T6SS1. The involvement of the T6SS2 in manganese acquisition and defense against oxidative stress likely promotes bacterial survival inside and outside the eukaryotic host (Si et al., 2017). Infection of *Galleria mellonella* indeed verified an important role of the T6SS2 in virulence *in vivo* (Si et al., 2017). Accordingly, we speculate that the virulence defect of the Δ*gvmR* mutant is in part caused by T6SS2 repression.

Altogether, in this study we identified a novel LTTR in *B. pseudomallei* that is essential for causing lethality of mice by coordinating expression of metabolic and virulence genes.

## Author Contributions

LD, KB, SS, IS, and CK conceived and designed the experiments. LD, KB, CK, FH, JM, and KE-P performed the experiments. CK, LD, FH, JM, and HG analyzed the data. SS, GW, IS, and CK wrote the paper. All authors read and approved the submitted version of the manuscript.

## Conflict of Interest Statement

The authors declare that the research was conducted in the absence of any commercial or financial relationships that could be construed as a potential conflict of interest.

## Footnotes

1 http://www.invitrogen.com

2 http://www.burkholderia.com

3 http://smart.embl-heidelberg.de

4 http://www.compbio.dundee.ac.uk/jpred/

5 http://www.genome.jp
