# Supplementary material for: GvmR – A Novel LysR-Type Transcriptional Regulator Involved in Virulence and Primary and Secondary Metabolism of Burkholderia pseudomallei
Source: Front Microbiol. 2018 May 16;9:935. doi: 10.3389/fmicb.2018.00935 (PMC5964159; doi:10.3389/fmicb.2018.00935)
Supplement: Supplementary file 1 [file Table_1.DOCX]

Table S1. List of primers used for quantitative real-time PCR.

| **Gene** | **Primer name** | **Sequence (5´ - 3´)** |
| --- | --- | --- |
| 23s RNA | 23s 1F | gtagacccgaaaccaggtga |
|  | 23s 1R | cacccctatccacagctcat |
| BPSL0492 | BPSL0492 1 F | catttcaagccgttcatgc |
|  | BPSL0492 1 R | cttcgatgccccatacctt |
| BPSL0937 | BPSL0937 1 F | CGACAACGAGATCAAGGACA |
|  | BPSL0937 1 R | GCTGCGTGTAGTACGTGTGC |
| BPSL1617 | BPSL1617 4 F | aggtattcccccgctatcac |
|  | BPSL1617 4 R | agtttccaggtctcgccttt |
| BPSL1778 | BPSL1778 1 F | atttcgcgtcggtgaactt |
|  | BPSL1778 1 R | cagcgtcgtgttcgtcac |
| BPSL1787 | BPSL1787 1 F | gttcgtgaagctcgtcgatt |
|  | BPSL1787 1 R | taggtgttctcgagcgtctg |
| BPSL2188 | BPSL2188 1 F | AGGAACTGCAAAAGCAATGG |
|  | BPSL2188 1 R | TTGTTGATGAGCGTCCAGAG |
| BPSL2289 | BPSL2289 5 F | GACAAGATGGTGCCGTATCTG |
|  | BPSL2289 5 R | CCCTTGCCCTGATAGAAGTG |
| BPSL2743 | BPSL2743 1 F | GATCTGGCCGAGAAGAAGGT |
|  | BPSL2743 1 R | ATCGGCTTGTCGGTGACTT |
| BPSL2974 | BPSL2974 1 F | AAGCGCAGCTGTTATTAGCC |
|  | BPSL2974 1 R | ATGTTGCCCGAACTGTTCTC |
| BPSL3036 | BPSL3036 1 F | gcccatacgtaccaggtgat |
|  | BPSL3036 1 R | gaagttgatttccgcgttgt |
| BPSS0005 | BPSS0005 5 F | cgctttcgctacaagaacaac |
|  | BPSS0005 5 R | atgccgtccatcgagaatac |
| BPSS0206 | BPSS0206 1 F | gcttcaaggcggtctatctg |
|  | BPSS0206 1 R | gtatcgatgtcgacgagcag |
| BPSS0310 | BPSS0310 1 F | taccctcatcgaacctgtcc |
|  | BPSS0310 1 R | tcctgcgtctgaatgatctg |
| BPSS0517 | BPSS0517 1 F | aggtggtcgtcgatcagaac |
|  | BPSS0517 1 R | tgttcgacacgaggtagtgc |
| BPSS0879 | BPSS0879 1 F | catcacgtaccaaagcaacg |
|  | BPSS0879 1 R | gctttccaacgtgaagatcg |
| BPSS1172 | BPSS1172 1 F | ggctgtcctatctgctcgac |
|  | BPSS1172 1 R | ggcgagaagaaactgtcctg |
| BPSS1173 | BPSS1173 1 F | ccgcactatctgctgaacaa |
|  | BPSS1173 1 R | gtgcagatcgagcttgtacg |
| BPSS1270 | BPSS1270 1 F | tatacggccgttcatctggt |
|  | BPSS1270 1 R | tcgacgatgtagctgtcgag |
| BPSS1496 | BPSS1496 1 F | agcgggtcaacatcgtctat |
|  | BPSS1496 1 R | acgtcgttgaagtcgtcctt |
| **Gene** | **Primer name** | **Sequence (5´ - 3´)** |
| BPSS1498 | BPSS1498 1 F | gtcatgacgggaaaatccac |
|  | BPSS1498 1 R | cgacgatctgtccatttcct |
| BPSS1522 | BPSS1522 1 F | aagcaggtgctcaagctgat |
|  | BPSS1522 1 R | tatcgatacgcccagtggat |
| BPSS1524 | BPSS1524 1 F | cgtgtacgagcacgtcagtt |
|  | BPSS1524 1 R | atcgccggaaaatagacctt |
| BPSS1529 | BPSS1529 1 F | ggactacatctcggccaaag |
|  | BPSS1529 1 R | atcagcttgtccggattgat |
| BPSS1533 | BPSS1533 1 F | cgctttctgtgcatctacga |
|  | BPSS1533 1 R | agataggactgcgccttctg |
| BPSS1638 | BPSS1638 1 F | tatccggcctgtatttcgag |
|  | BPSS1638 1 R | gtcgacgagcagtttcttcc |
| BPSS1955 | BPSS1955 4 F | cgaccagacgctcgagtt |
|  | BPSS1955 4 R | cagcacgatccgcttttc |
| BPSS2000 | BPSS2000 1 F | acagcctgacgctcaagaat |
|  | BPSS2000 1 R | tgtagacgtcgacctgcttg |
|  |  |  |
