# Supplementary material for: GvmR – A Novel LysR-Type Transcriptional Regulator Involved in Virulence and Primary and Secondary Metabolism of Burkholderia pseudomallei
Source: Front Microbiol. 2018 May 16;9:935. doi: 10.3389/fmicb.2018.00935 (PMC5964159; doi:10.3389/fmicb.2018.00935)
Supplement: Supplementary file 3 [file Table_3.DOCX]

**Table S3.** Comparison of the proteins encoded by the *afc* region of *B. cenocepacia* J2315 with those of the *afc* region of *B. pseudomallei* K96243.

| protein of *B.c.* J2315  old designation / new designation | proposed function | orthologous protein of *B.p.* K96243 | AAI % |
| --- | --- | --- | --- |
| BCAS0201 / WP_012493693 (AfcF) | putative FAD-dependent oxidoreductase | o.p.o. | n.a. |
| BCAS0202 / WP_006491295 | permease | BPSL0477 | 39 |
| BCAS0203 / WP_012493694 | ABC-transporter | BPSL0478 | 57 |
| BCAS0204 / WP_020980038 | ABC transporter ATP-binding protein | BPSL0479 | 53 |
| BCAS0205 / WP_006490906 | TauD/TfdA taurine catabolism dioxygenase | BPSL0480 | 54 |
| BCAS0206 / WP_006490905 | SAM-dependent methyl-transferase | BPSL0481 | 74 |
| BCAS0207 / WP_012493696 | citrate synthase | BPSL0482 | 55 |
| BCAS0208 / WP_006482087 (AfcE) | putative acyl-CoA dehydrogenase | BPSL0483 | 70 |
| BCAS0209 / WP_012493698 | 3-oxoacyl-ACP synthase III | BPSL0484 | 45 |
| BCAS0210 / WP_006487272 | AMP-binding enzyme | BPSL0485 | 63 |
| BCAS0211 / WP_006493646 | putative pyridoxal-dependent decarboxylase | BPSL0486 | 54 |
| BCAS0212 / WP_006487266 | condensing enzymes (Claisen condensation) | BPSL0487 | 54 |
| BCAS0213 / WP_085964412 | ferritin like protein | BPSL0488 | 76 |
| BCAS0214 / WP_006487268 | hypothetical protein | BPSL0489 | 39 |
| BCAS0215 / WP_006493647 | putative exported protein | BPSL0490 | 50 |
| BCAS0216 / WP_006481176 | putative Acyl Carrier Protein (ACP) | BPSL0491 | 81 |
| BCAS0217 / WP_006482239 | hypothetical protein | BPSL0492 | 56 |
| BCAS0218 / WP_012493699 | hypothetical protein | no hit | 0 |
| BCAS0219 / WP_006482245 | outer membrane lipoprotein-sorting | BPSS2323^a^ | 26 |
| BCAS0220 / WP_006482237 | ABC-transporter – permease component | BPSS2324^a^ | 29 |
| BCAS0221 (encoded by frame shifted pseudo gene) | ABC transporter | BPSS2325^a^ | n.a. |
| BCAS0222 / WP_034178506 (AfcA) | putative AMP-dependent synthase | BPSL0493 | 51 |
| BCAS0223 / WP_006482247 (AfcC) | putative omega-3 FA desaturase | BPSL0475 | 33 |
| BCAS0224 / WP_034178508 (AfcD) | ferritin-like protein | o.p.o. | n.a. |
| BCAS0225 / WP_006482230 (ShvR) | LysR family transcriptional regulator (shiny morphotype) | BPSL0494 | 25 |

^a^located on chromosome 2 instead of Chr 1. Abbeviations: AAI % = percentage of amino acid identity; o.p.o. = only paralogs observed ; n.a. = not applicable.
