# Supplementary material for: GvmR – A Novel LysR-Type Transcriptional Regulator Involved in Virulence and Primary and Secondary Metabolism of Burkholderia pseudomallei
Source: Front Microbiol. 2018 May 16;9:935. doi: 10.3389/fmicb.2018.00935 (PMC5964159; doi:10.3389/fmicb.2018.00935)
Supplement: Supplementary file 4 [file Presentation_1.PDF]

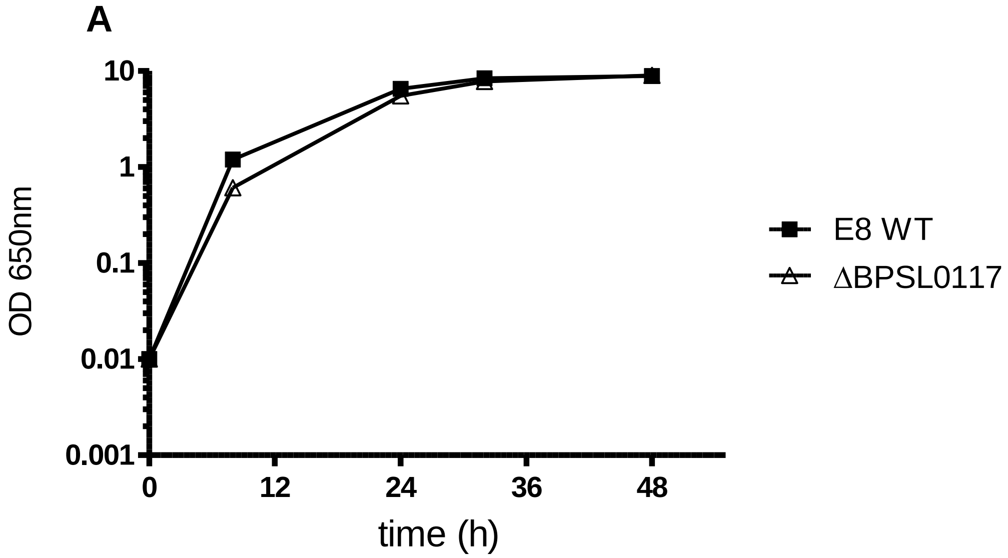

Figure S1. Growth of *B. pseudomallei* wild type and Δ*gvmR* mutant. Bacteria were grown in LB medium at 37°C and 140 rpm for 48 hours. Shown are mean values of two independent experiments. Error bars indicate standard error of the mean (SEM).

Figure S2 A-D. Quantitative RT-PCR (qRT PCR) validation of tiling microarray results of selected genes representing functional categories described in the text. Gene expression differences in the Δ*gvmR* mutant measured by qRT-PCR and microarray (array) are represented as fold change relative to the *B. pseudomallei* wild type (wt). Data from qRT PCR experiments were normalized using the 23S rRNA gene as internal control. Shown are mean values of three independent experiments. Error bars indicate standard error of the mean (SEM). **A**: Genes involved in core metabolism pathways. **B**: Genes of unknown function. **C**: Genes representing secondary metabolism pathways. **D**: Virulence-associated genes involved in T3SS3, T6SS1 and T6SS2.

BPSS0879 - Outer membrane protein (porin); BPSS1498 - type VI secretion system effector, Hcp1 family; BPSS1270 - Uncharacterized protein conserved in bacteria; BPSS1533 type III secretion low calcium response chaperone LcrH/SycD; BPSS0005 -2-amino-3-ketobutyrate coenzyme A ligase (EC 2.3.1.29); BPSS1522 - two component transcriptional regulator, LuxR family; BPSL0492 - hypothetical protein; BPSS1529 - type III secretion system translocon protein, IpaD/SipD family; BPSS1524 - SicP binding; BPSS1496 - type VI secretion protein, VC_A0107 family; BPSS0517 - type VI secretion protein, EvpB/VC_A0108 family; BPSL2289 - cysteine desulfurase IscS; BPSS1638 - Acyl dehydratase; BPSL2188 - isocitrate lyase (EC 4.1.3.1); BPSS1173 - non-ribosomal peptide synthase/polyketide synthase; BPSS0206 - methylisocitrate lyase (EC 4.1.3.30); BPSL1787 - RNA polymerase sigma factor, sigma-70 family; BPSL1778 - siderophore related no-ribosomal peptide synthase; BPSS1172 - non-ribosomal peptide synthase/polyketide synthase; BPSL1617 - hypothetical protein (flavoproteins); BPSS2000 - Cupredoxin-like domain protein; BPSS1955 - bifunctional enoyl-CoA hydratase/phosphate acetyltransferase; BPSL0937 - Ribulose-5-phosphate 4-epimerase and related epimerases and aldolases; BPSL2743 - Zn-dependent hydrolases, including glyoxylases; BPSS0310 - hypothetical protein; BPSL3036 - Outer membrane protein (porin); BPSL2973 - hypothetical protein

Figure S2A

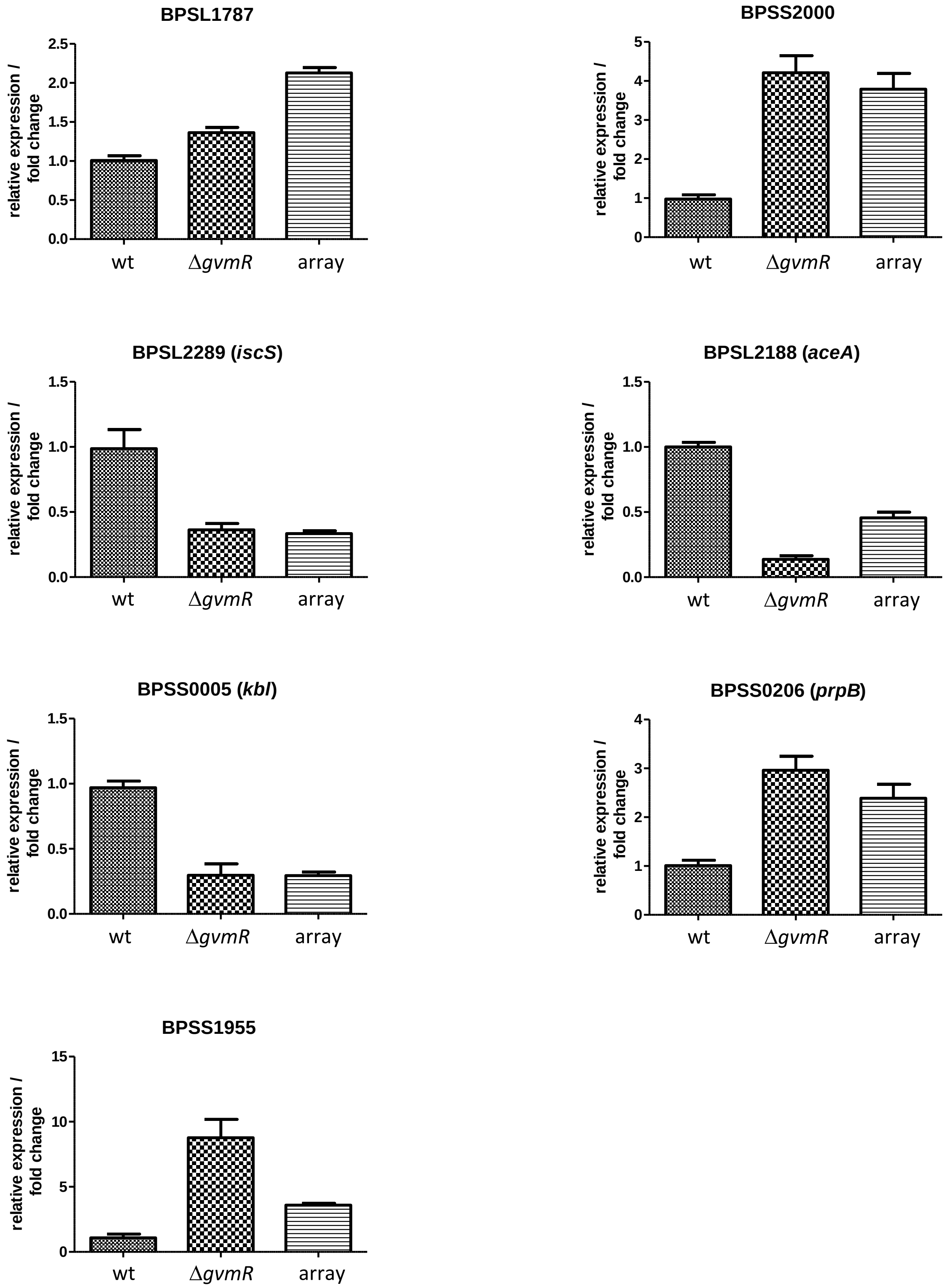
BPSL1787
relative expression / fold change
2.5
2.0
1.5
1.0
0.5
0.0
wt
ΔgvmR
array
BPSS2000
relative expression / fold change
5
4
3
2
1
0
wt
ΔgvmR
array
BPSL2289 (iscS)
relative expression / fold change
1.5
1.0
0.5
0.0
wt
ΔgvmR
array
BPSL2188 (aceA)
relative expression / fold change
1.5
1.0
0.5
0.0
wt
ΔgvmR
array
BPSS0005 (kbl)
relative expression / fold change
1.5
1.0
0.5
0.0
wt
ΔgvmR
array
BPSS0206 (prpB)
relative expression / fold change
4
3
2
1
0
wt
ΔgvmR
array
BPSS1955
relative expression / fold change
15
10
5
0
wt
ΔgvmR
array

Figure S2B

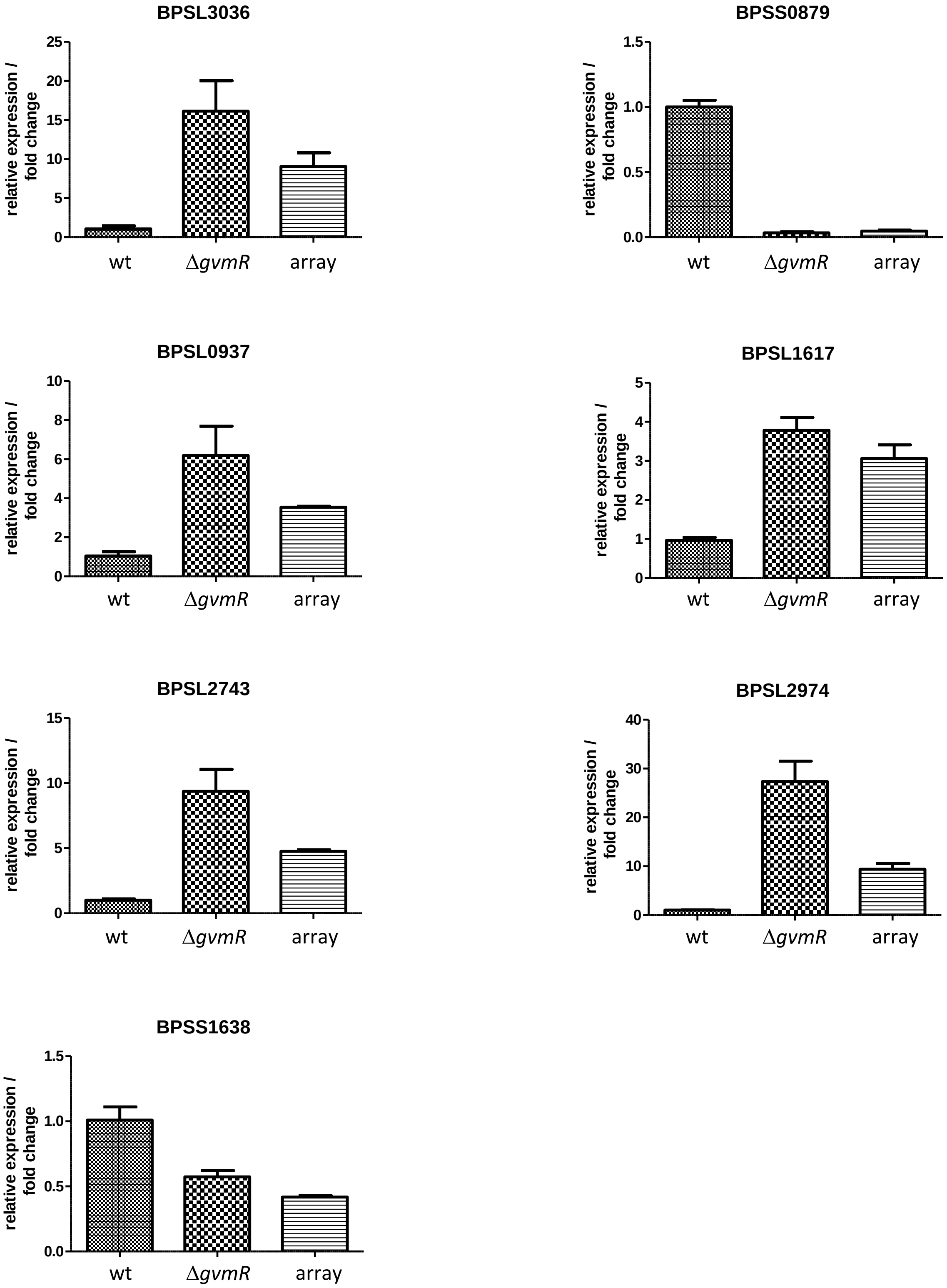
BPSL3036
relative expression / fold change
0
5
10
15
20
25
wt
ΔgvmR
array
BPSS0879
relative expression / fold change
0.0
0.5
1.0
1.5
wt
ΔgvmR
array
BPSL0937
relative expression / fold change
0
2
4
6
8
10
wt
ΔgvmR
array
BPSL1617
relative expression / fold change
0
1
2
3
4
5
wt
ΔgvmR
array
BPSL2743
relative expression / fold change
0
5
10
15
wt
ΔgvmR
array
BPSL2974
relative expression / fold change
0
10
20
30
40
wt
ΔgvmR
array
BPSS1638
relative expression / fold change
0.0
0.5
1.0
1.5
wt
ΔgvmR
array

Figure S2C

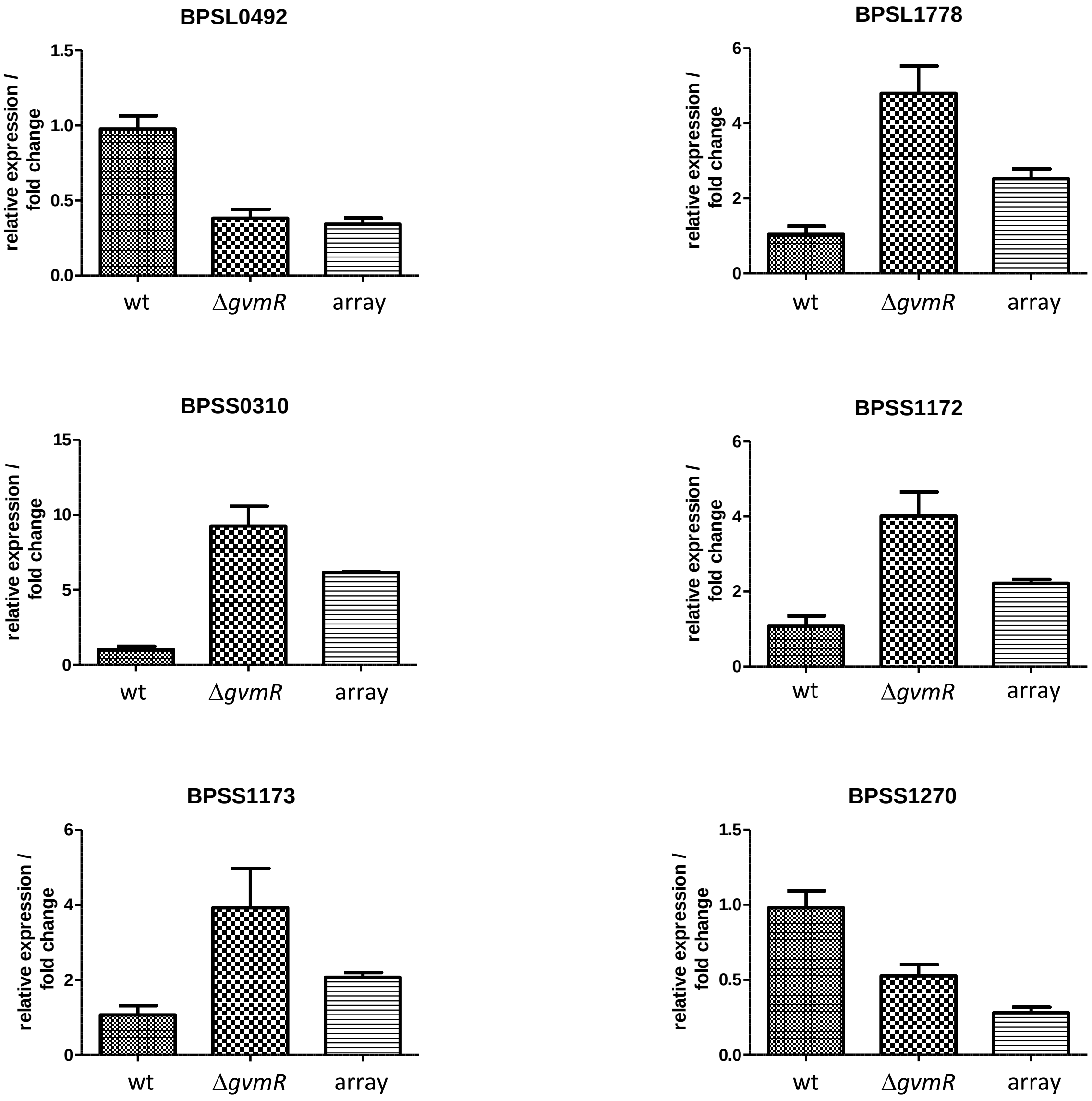
BPSL0492
relative expression / fold change
1.5
1.0
0.5
0.0
wt
ΔgvmR
array
BPSL1778
relative expression / fold change
6
4
2
0
wt
ΔgvmR
array
BPSS0310
relative expression / fold change
15
10
5
0
wt
ΔgvmR
array
BPSS1172
relative expression / fold change
6
4
2
0
wt
ΔgvmR
array
BPSS1173
relative expression / fold change
6
4
2
0
wt
ΔgvmR
array
BPSS1270
relative expression / fold change
1.5
1.0
0.5
0.0
wt
ΔgvmR
array

Figure S2D

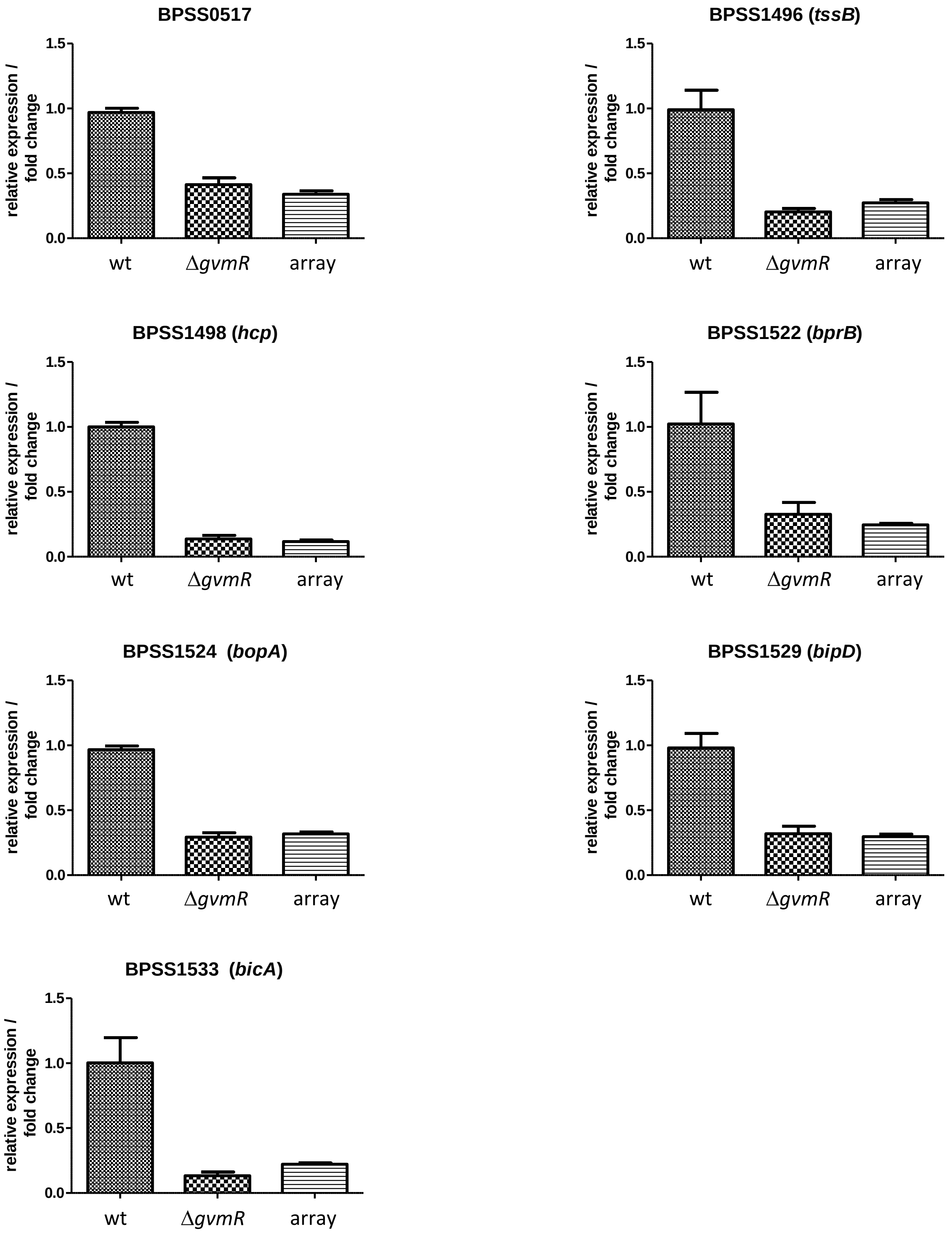

BPSS0517
BPSS1496 (tssB)
BPSS1498 (hcp)
BPSS1522 (bprB)
BPSS1524 (bopA)
BPSS1529 (bipD)
BPSS1533 (bicA)
relative expression / fold change
0.0
0.5
1.0
1.5
wt
ΔgvmR
array

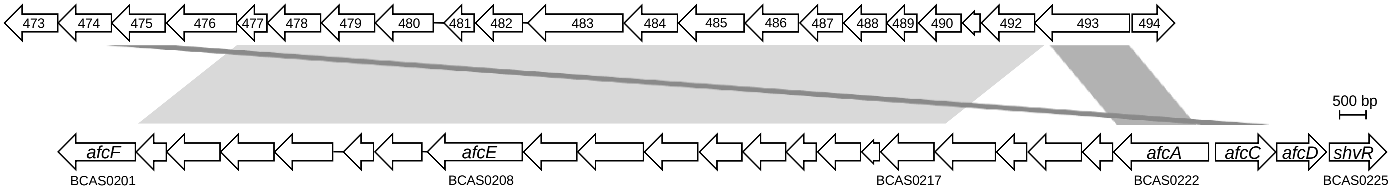

Figure S3. Synteny map of the *B. pseudomallei* K96243 gene cluster BPSL0473-0493 (top) and the *afc* region of *B. cenocepacia* J2315 (bottom). The gray bands connect conserved genes of the two clusters. ORFs of *B. pseudomallei* with similarity to BCA0219, BCAS0220 and BCAS0221 are located outside of the BPSL0473-0493 gene cluster (Table S3). No orthologs could be found for BSAC0218, *afcF* and *afcD* (Table S3).
